# Distal jejunal gallstone ileus—an unusual cause of bowel obstruction: a case report

**DOI:** 10.1093/jscr/rjad557

**Published:** 2023-10-17

**Authors:** Munaser Alamoodi

**Affiliations:** Department of Surgery, Faculty of Medicine, King Abdulaziz University, 3239 Suliaman Abdullah Suliaman street, Jeddah 21589, Saudi Arabia

**Keywords:** gallstones ileus, small bowel obstruction, distal jejunum, enterotomy, enterolithotomy

## Abstract

Gallstone ileus is a rare entity that causes bowel obstruction by gaining access through a cysto-enteric fistula. This is a case report of a 70-year-old man presenting with small bowel obstruction secondary to distal jejunal gallstone ileus impaction. He is not known to have any predisposing factors. He was managed effectively with an enterolithotomy. Early diagnosis is key to a good prognosis. Although other management options are available, enterolithotomy with or without cholecystectomy remains the gold standard of management.

## Introduction

Gallstone ileus is a rare entity, often occurring in the elderly population, and accounts for <1% of mechanical bowel obstructions. It occurs as a result of a cholecysto-enteric fistula. This episode is often preceded by acute cholecystitis. The diagnosis is challenging and should be suspected in the elderly who present with abdominal distension in a virgin abdomen.

## Case report

This is the case of a 70-year-old man who presented to the ER with a 2-day history of abdominal pain, distension, and vomiting. The episode began with a 1-day history of right upper quadrant pain. He has no other medical or surgical history and has never been diagnosed with gallstones.

On examination, his vitals were stable. His abdomen was distended, soft, and non-tender. Bowel sounds were absent. The lab investigations were all within the normal range.

**Figure 1:**
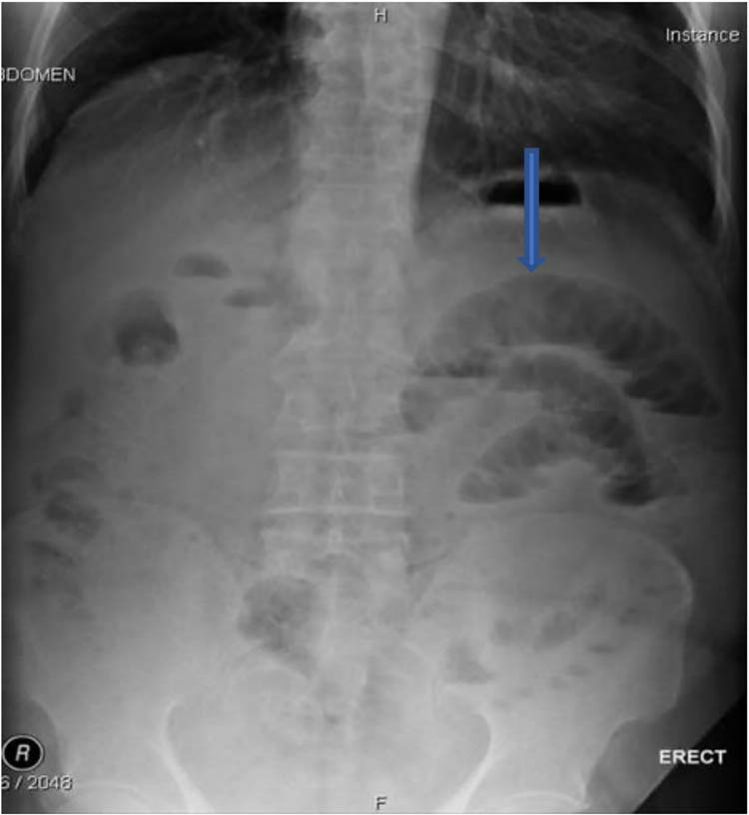
Erect film showing air-fluid levels.

**Figure 2:**
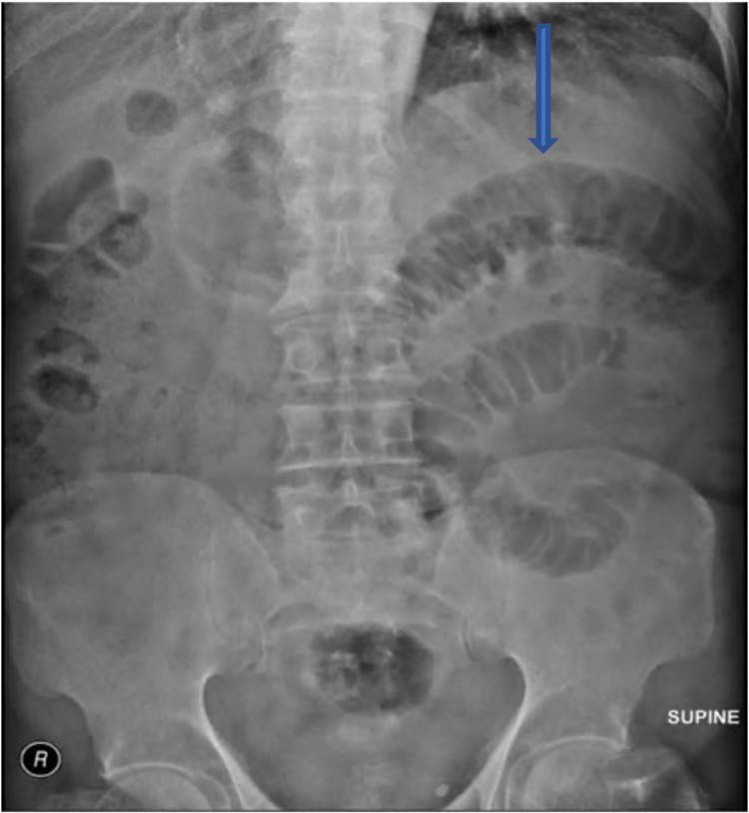
Supine film with small bowel dilatation.

**Figure 3:**
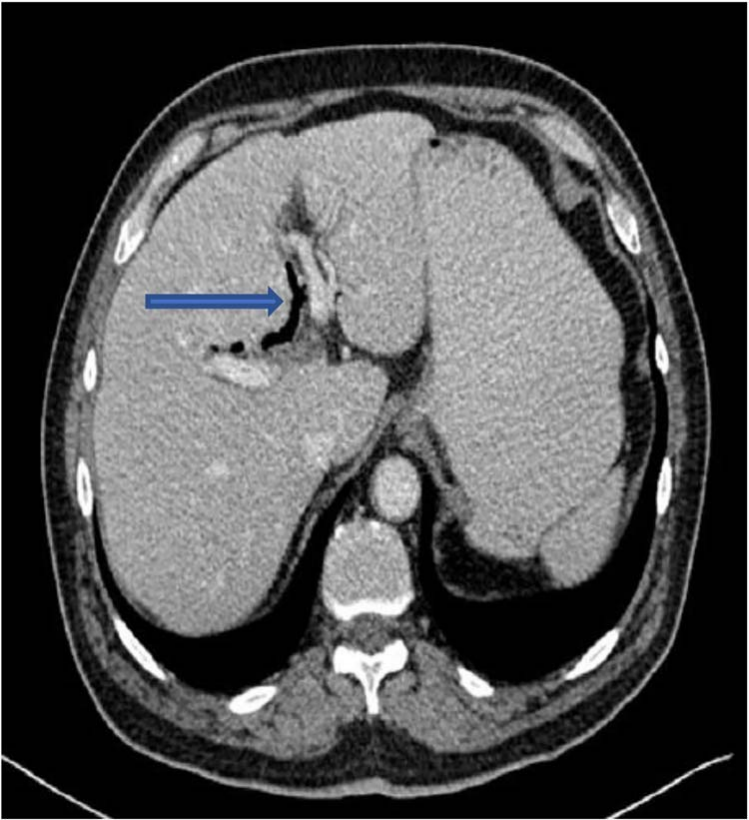
CT showing pneumobilia.

**Figure 4:**
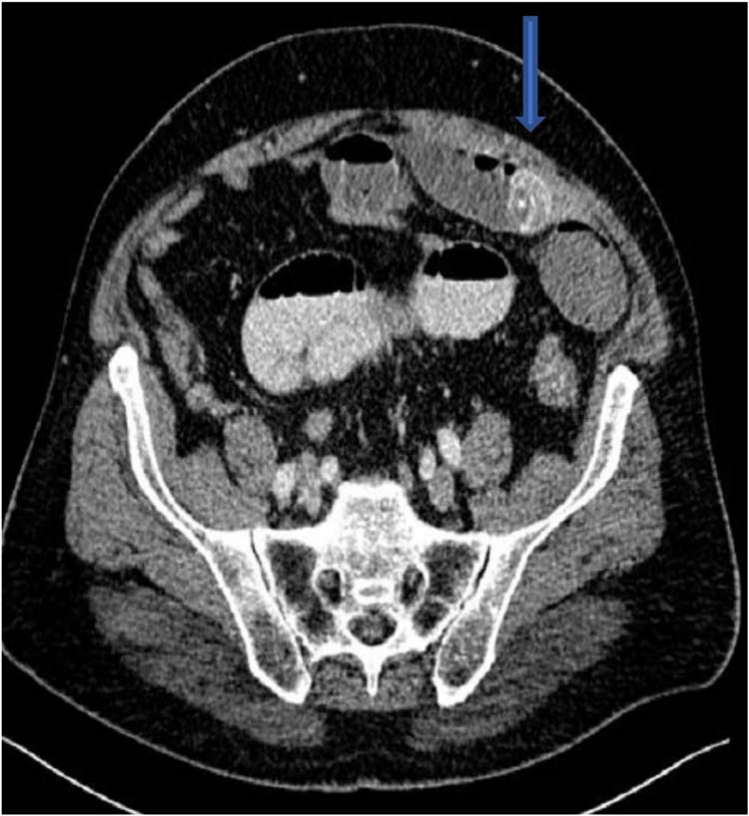
CT showing stone in distal jejunum.

The plain film of the abdomen showed multiple air-fluid levels on the erect film (Fig. 1) and small bowel dilatation on the supine (Fig. 2). The CT showed multiple air pockets in the biliary tree, representing pneumobilia (Fig. 3). It also showed evidence of a circular radio-dense stone measuring 2 cm within the distal jejunum, leading to obstruction and dilatation of the proximal jejunum (Fig. 4).

**Figure 5:**
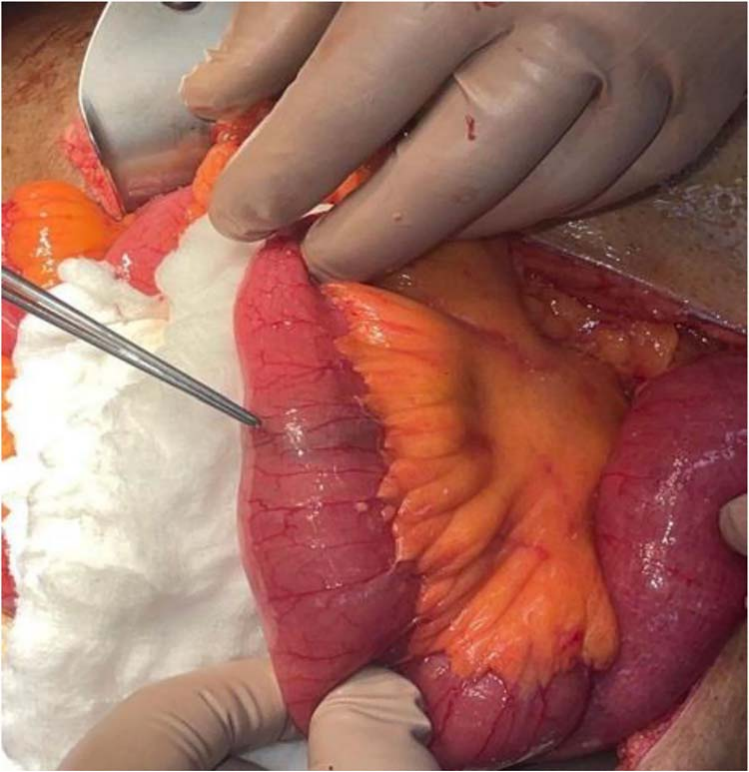
Stone in distal jejunum.

**Figure 6:**
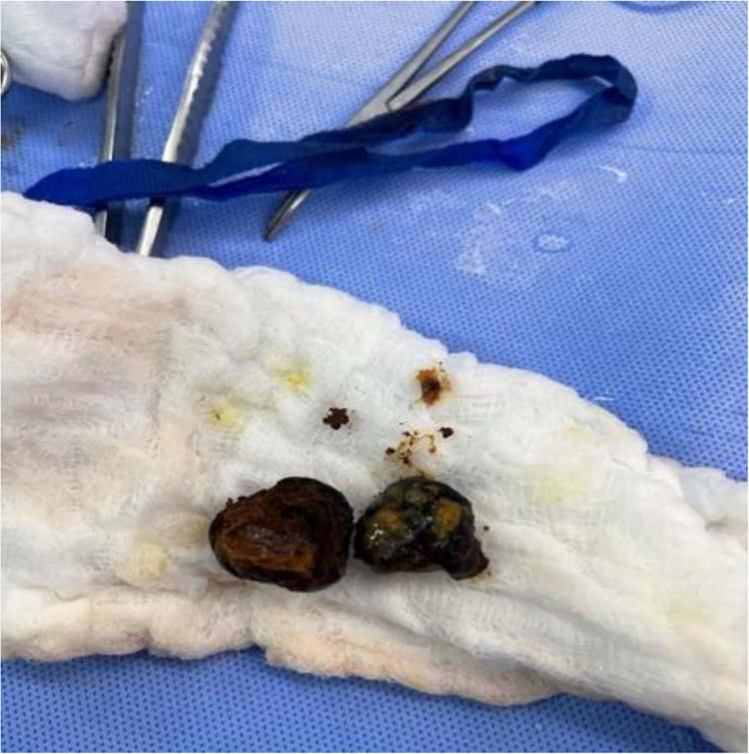
retrieved stone in two pieces with proximal dilatation.

The patient was taken to the operating theater, and an upper midline incision laparotomy was carried out. The site of the foreign body (stone) was identified in the distal jejunum (Fig. 5), and an enterotomy was performed longitudinally. The stone was retrieved in two pieces (Fig. 6), and the enterotomy was closed transversally. The patient was discharged home on day three post-op. He was followed up in the clinic two weeks later and was doing well with no further events.

## Discussion

Although gallstone ileus is an uncommon cause of small bowel obstruction, it accounts for 1%–4% of all intestinal obstructions [1]. Early diagnosis could be challenging and is a key factor in decreasing morbidity and mortality. The mortality ranges 12%–18%, as most patients are elderly and have comorbidities [2].

Gallstone ileus occurs in 0.3%–0.5% of patients with cholelithiasis [3], and it accounts for 25% of mechanical obstruction of the small bowel in patients over the age of 65 [4]. The stone gets access through a fistula between the gallbladder and part of the bowel. Commonly, it gains access through a cholecysto-duodenal fistula in 68% of patients with gallstone ileus [5]. It can rarely gain access without a fistula by means of a stone passing through the ampulla of Vater, followed by *in-situ* growth [6]. Once it gains access, it can lodge in any part of the bowel. The terminal ileum is the commonest site, being the narrowest [1]. The second-commonest is the jejunum, at 30% [7]. Less commonly, lodgment of the stone in the duodenum (3%–10%) gives rise to Bouveret’s syndrome, leading to gastric outlet obstruction [3].

CT is the investigation of choice. Plain films can reveal dilated small bowels, confirming small bowel obstruction [8]. The presence of Rigler’s triad on the plain film, which includes pneumobilia, dilated bowel, and ectopic gallstones, is diagnostic of gallstone ileus, but this occurs only in 9%–14% of patients [7]. Most patients require surgical intervention to relieve the obstruction. Surgery includes enterotomy and removal of the stone (enterolithotomy) or enterolithotomy with cholecystectomy and repair of the fistula [2]. Commonly, simple enterolithotomy is favored as it carries less morbidity, with or without cholecystectomy at a later date [9]. Spontaneous fistula closure occurs in up to 50% of cases [7].

Another surgical option that also carries lower morbidity is laparoscopic retrieval of the stone; this, however can be challenging due to the difficulty faced when manipulating the distended bowel and requires special expertise. Non-invasive management, such as endoscopic retrieval of the obstructing stone, could also be an option in selected patients [10].

## Conclusion

Gallstone ileus should be suspected in elderly patients who present with small bowel obstruction and have had no previous abdominal surgery. The procedure of choice remains a simple enterolithotomy. Laparoscopic retrieval of the stone could be an option.
